# Na^+^/H^+^ Exchanger 1, a Potential Therapeutic Drug Target for Cardiac Hypertrophy and Heart Failure

**DOI:** 10.3390/ph15070875

**Published:** 2022-07-15

**Authors:** Huiting Xia, Aqeela Zahra, Meng Jia, Qun Wang, Yunfu Wang, Susan L. Campbell, Jianping Wu

**Affiliations:** 1 School of Chemistry, Chemical Engineering and Life Sciences, Wuhan University of Technology, Wuhan 430070, China; xiaht@whut.edu.cn (H.X.); Zahra@whut.edu.cn (A.Z.); 2 Beijing Tiantan Hospital, Capital Medical University, Beijing 100070, China; jiameng@bjtth.org (M.J.); wangq@ccmu.edu.cn (Q.W.); 3 Advanced Innovation Center for Human Brain Protection, Capital Medical University, Beijing 100070, China; 4 National Clinical Research Center for Neurological Disease, Beijing 100070, China; 5 Taihe Hospital, Hubei University of Medicine, Shiyan 440070, China; wyfymc@163.com; 6 Animal and Poultry Sciences, Virginia Polytechnic Institute and State University, Blacksburg, VA 24060, USA; susanc08@vt.edu

**Keywords:** Na^+^/H^+^ exchanger 1, cardiac hypertrophy, heart failure, ischemia-reperfusion injury

## Abstract

Cardiac hypertrophy is defined as increased heart mass in response to increased hemodynamic requirements. Long-term cardiac hypertrophy, if not counteracted, will ultimately lead to heart failure. The incidence of heart failure is related to myocardial infarction, which could be salvaged by reperfusion and ultimately invites unfavorable myocardial ischemia-reperfusion injury. The Na^+^/H^+^ exchangers (NHEs) are membrane transporters that exchange one intracellular proton for one extracellular Na^+^. The first discovered NHE isoform, NHE1, is expressed almost ubiquitously in all tissues, especially in the myocardium. During myocardial ischemia-reperfusion, NHE1 catalyzes increased uptake of intracellular Na^+^, which in turn leads to Ca^2+^ overload and subsequently myocardial injury. Numerous preclinical research has shown that NHE1 is involved in cardiac hypertrophy and heart failure, but the exact molecular mechanisms remain elusive. The objective of this review is to demonstrate the potential role of NHE1 in cardiac hypertrophy and heart failure and investigate the underlying mechanisms.

## 1. Introduction

The primary function of the heart is to pump blood to all the organs throughout the body, and to fulfil demands under either normal or stressful conditions. Hypertrophy occurs when the heart or the cardiomyocytes enlarge in response to increased preload or afterload. The heart initially responds compensatory to the cardiac injury by increasing the size and mass of the heart, normalizing ventricular wall stress to maintain cardiac function [1]. This type of cardiac hypertrophy is usually termed pathological cardiac hypertrophy. However, over time in settings of sustained workload, chronic stress or injury will contribute to left ventricular dilation, ultimately leading to heart failure [2].

Heart failure is a complex clinical syndrome induced by defective cardiac anatomy and/or function [3]. It remains a major public health concern, with prevalence expected to rise by 46% from 2012 to 2030, contributing to over 8 billion American adults suffering from heart failure. In the United States, roughly 50% of heart failure cases occur in 6% of the population over the age of 70 [4,5]. In elderly patients with chronic heart failure, insufficient energy, increased energy expenditure, and anabolic dysfunction collectively lead to malnutrition which seems to mediate disease progression. Deficiencies in both macronutrients and micronutrients may lead to the wasting process once triggered [6,7]. Deficiency in micronutrients as a cause of heart failure is rare, but cases due to selenium deficiency have been reported [8]. In a study investigating serum selenium levels as a predictor of mortality in individuals aged over 80 years, it has been demonstrated that low selenium concentrations may be associated with mortality [9]. Additionally, elderly patients with heart failure are at higher risk of potential drug-drug interactions due to polypharmacy, accompanied by associated effects including poor compliance and adverse events, and deprescription may reduce the burden of treatment and mortality in the elderly [10,11]. Although there have been substantial improvements in treating heart failure, the incidence of heart failure is increasing rapidly, posing a severe threat to human health. A wide variety of heart diseases, genetic defects, as well as systemic diseases can contribute to heart failure. Patients with heart failure may have a combination of etiologies that are not mutually exclusive. However, the definite pathogenesis of heart failure remains unknown. Ischemic heart disease, usually caused by acute or chronic myocardial ischemia, is responsible for the majority of heart failure cases [12]. Timely myocardial reperfusion, either by thrombosis or angioplasty, is the only treatment recognized as being capable of saving the ischemic myocardium and improving the patient’s prognosis [13]. Before the advent of thrombolytic therapy, it was discovered that restoring the blood supply (which is necessary to remedy oxygen-starved issues that did not develop irreversible damage) could paradoxically exacerbate ischemic damage and further increase infarct size. Both the extent of reduced blood flow and the duration of ischemia affects the degree of cellular dysfunction and mortality [14]. Using a three-dimensional oxygen reperfusion model, it has been demonstrated that reperfusion is beneficial despite the risk of developing myocardial injury [15]. Since the second half of the twentieth century, cardiovascular researchers have focused on the mechanisms that lead to myocardial injury during ischemia-reperfusion. The current therapeutic arsenal against ischemic heart disease includes drugs that slow the progression of atherosclerosis, treatments that restrain intracoronary thrombosis, and drugs and therapies that re-establish coronary reperfusion [16]. Nevertheless, the strategies lack direct cardioprotective agents which specifically target cardiomyocytes. Therefore, there is an urgent need for safe and effective new therapies capable of retarding the development of myocardial ischemia and/or mitigating the deleterious consequences of reperfusion.

The Na^+^/H^+^ exchangers (NHEs) simultaneously transport one Na^+^ into the cells and one H^+^ out of the cells, driven by the energy in the form of electrochemistry Na^+^ concentration gradients [17]. The most well-studied subtype, NHE1, is a ubiquitously expressed plasma membrane protein that regulates many cellular functions due to its multiple housekeeping tasks, such as regulating intracellular pH (pH_i_), Na^+^ concentration, and cell volume in almost all tissues. NHE1 is the only cardiac-specific plasma membrane isoform of the NHE family. In the heart, the exacerbated activity of NHE1 has been associated with pathological cardiac processes. In several animal models which reproduce the detrimental effects of ischemia-reperfusion injury (IRI) or cardiac hypertrophy, NHE1 inhibition is demonstrated to be cardioprotective [18]. Extensive studies have investigated the role of NHE1 in cardiac hypertrophy and heart failure, which indicates that NHE1 may serve as a potential drug target. In this review, we describe the structure and physiological functions of NHE1 and emphasize its role in myocardial ischemia-reperfusion injury and cardiac hypertrophy, the underlying mechanism of which has also been discussed.

## 2. Distribution and Structure of NHE1

### 2.1. NHE1 Distribution

Mitchell et al. [19] were the first to hypothesize the presence of an electroneutral transport mechanism for cation and H^+^ to exchange across the mitochondrial inner membrane. Subsequently, several studies verified the activity of NHEs in the bacterium, which was later described in mammals [20].

NHEs are encoded by the SLC9 gene family of the solute carrier (SLC) transporters [21]. Three subfamilies of the SLC9 gene family have been identified based on phylogenetic analysis. The SLC9A subgroup comprises nine mammalian NHE paralogs, NHE1-9. The SLC9B subgroup includes two Na^+^ or Li^+^/H^+^ antiporters, NHA1 (SLC9B1) and NHA2 (SLC9B2). The SLC9C subgroup consists of NHE10 (SLC9C1), expressed primarily in sperm tissue and osteoblast, and an orphan-related protein NHE11 (SLC9C2) [20,21,22,23].

The members of the mammalian SLC9A gene family are divided into plasma membrane clusters and intracellular organelle clusters. The NHE family members NHE1-5 are primarily found in the plasma membranes, while NHE6-9 localize to the organelle chambers [24]. Among the plasma membrane clusters, NHE1 is ubiquitous in the plasma membrane of almost all tissues, particularly the heart, the brain, and the peripheral nervous system [24]. Even though NHE1 is typically localized to the basolateral membrane of diverse epithelia, it is also found in the apical membrane of choroid plexus epithelia. In cardiomyocytes, NHE1 is located in intercalated disks and T-tubules rather than in the peripheral sarcolemmal membranes, which may influence local pH [21].

### 2.2. NHE1 Structure

**Figure 1:**
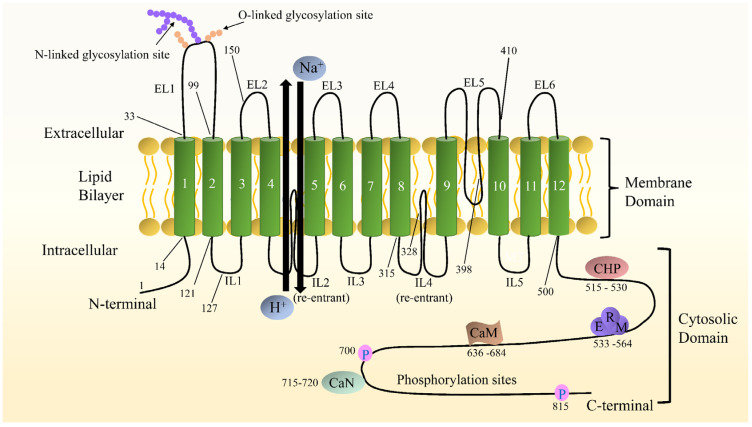
Schematic diagram of NHE1 topological structure. Both the N- and C- termini are located in the cytoplasm. The numbers indicate predicted transmembrane domains. Some representative amino acids are pointed out. The C-terminal hydrophilic cytoplasmic structural domain contains binding sites for a variety of proteins, including calmodulin (CaM), calcineurin (CaN), calcineurin homologous protein (CHP), and esrin/radixin/moesin (ERM), as well as cytoplasmic structural domains involved in phosphorylation and activity regulation.

A detailed insight into the structure of NHE1 is imperative for understanding its mechanisms and developing more powerful inhibitors. Nevertheless, the high-resolution structure of NHE1 remains elusive due to difficulties in crystallizing the protein molecules. The primary structure of human NHE1 was first described by Claude Sardet et al. They predicted that human NHE1 had 815 amino acids, with the first 500 residues comprising 10 transmembranes (TMs) helixes, and the hydrophilic C-terminal tail of 315 residues comprising a regulatory domain, based on hydropathy analysis [25]. Shrode et al. [26] confirmed the intracellular location of the NH2 and COOH termini via chymotryptic cleavage and epitope tagging. Moreover, the region between TM9–10 and TM11–12 was not completely available from the outside of the cells, suggesting that it might be buried inside the membrane. Based on experimentation using substituted cysteine accessibility examination, Wakabayashi et al. [27] proposed a novel model for the topology of NHE1 with the N- and C-termini situated in the cytol and the N-glycosylation site at N75. Na^+^ and H^+^ exchange is carried out by 12 relatively conserved TM segments joined by short loops and two long re-entrant loops, while 315 residues compose a more divergent hydrophilic C-terminal cytosolic domain that plays a regulatory role, and 15 residues form an extremely short hydrophobic N-terminal tail that extends into the cell [20]. Additionally, there are several binding sites in the C-terminal domain, such as esrin/radixin/moesin (ERM), calmodulin (CaM), calcineurin (CaN), and calcineurin homologous protein (CHP) [28]. The model of NHE1 is shown in Figure 1. The greatest potential breakthrough stems from elucidating the high-resolution structures of the bacterial Na^+^/H^+^ antiporters, including the well-known antiporter *Escherichia coli* (*Ec*)NhaA, which are easy to overproduce and crystallize. The bacterial *Ec*NhaA and eukaryotic Na^+^/H^+^ exchanger play similar roles in the regulation of pH_i_ and electrolyte homeostasis and thus are considered to share a similar structural folding [22,29]. Later, Landau et al. developed a novel topological model of NHE1 [30]. It was obtained computationally, including a revolutionary conserved analysis and a folding comparison with NhaA [31], comparable to Wakabayashi’s model with 12 TM domains, although amino acids 1-125 were considered to be eliminated by cleavage. In this model, the TM9 (amino acid 341-362) of Wakabayashi’s model was redistributed into two helices, TM7 and TM8, and the re-entrant segment extracellular loop (EL) 5 was reassigned to TM9. The last three TMs are identical in these two models [29]. Liu et al. [32] used cysteine scanning accessibility and detection of glycosylation of mature proteins to characterize the NHE1 protein, and they demonstrated that the basic features of Wakabayashi were correct. The latest three-dimensional model of human NHE1 was predicted based on the known structure of *Methanocaldoccocus jannaschii* (more similar in sequence and function to mammalian SLC9As than *Ec*NhA) and combined with biochemical surface accessible data [33]. The specific model description was reviewed in Dutta’s [29].

## 3. NHE1 in Cardiac Physiology Regulation

NHEs are the most extensively studied acid-base regulators in various mammalian cells, including cardiomyocytes. They act a pivotal part in regulating cytoplasmic acid-base homeostasis together with HCO_3_^−^ transport systems, including Na^+^/HCO_3_^−^ cotransporters (NBCs) and Cl^−^/HCO_3_^−^ anion exchangers (AEs) [34]. In many cell types, NHE1 is the primary alkalinization mechanism, preventing the destructive effects of excessive acidification. The intracellular acid load generated by normal cardiomyocyte metabolism activates NHE1 protein to transport one intracellular proton inside the cells and one extracellular Na^+^ outside the cells, thereby preventing cellular acidosis. In this process, the inward Na^+^ gradient created by the Na^+^/K^+^ ATPase drives the counter transport of proton from the cytoplasm [35]. Furthermore, NHE1 is also engaged in regulating cardiomyocyte volume, growth, proliferation, apoptosis, and differentiation, as well as a series of physiological activities.

Cell volume is an important component of an organism’s homeostasis. Volume regulation is crucial to cardiomyocyte function in healthy and disease states. Fundamental metabolic processes, such as respiration, constantly produce osmotic active products, which, if not regulated, would flow into the cell, leading to its swelling [36]. In addition, various environmental conditions can contribute to alterations in intracellular and intravascular volume. Swelling or shrinkage of cells may weaken the integrity of the cell membrane. It is hypothesized that the shrinkage of cells in response to a hyperosmotic extracellular milieu may be counterbalanced by an increased influx of Na^+^ and Cl^−^, accompanied by the osmolarity-driven water influx and compensatory swelling. Known as regulatory volume increase (RVI), this process serves to counteract the decrease of cell volume [20,36]. Other transporters have also been involved in RVI, including Na^+^/K^+^/2Cl^−^ cotransporters (NKCC), electroneutral anion exchanger Cl^−^/HCO_3_^−^ (AE2) [20]. The H^+^ excretion through NHEs and HCO_3_^−^ exiting through AE2 are supplemented in the cell by H_2_CO_3_, which is easily produced from CO_2_, the product of aerobic metabolism. This process causes the entry of NaCl. On the other hand, Na^+^ entering the cells through NKCC and NHEs is pumped out by Na^+^/K^+^ ATPase to exchange K^+^, which ultimately results in the uptake of KCl by the cells [28]. In addition, the NKCC isoforms NKCC1 and NKCC2 and NHE isoforms NHE1, 2, and 4 are shown to be contraction-activated, whereas NHE3 and 5 are manifested as contraction-inhibited [35,37]. It has been demonstrated that NHE1 activation caused by cell contraction depends on the activity of CaM, which is likely downstream of tyrosine kinase Janus kinase 2(JAK2) and requires active phospholipase C (PLC).

The progression of the cell cycle is hypersensitive to cytoplasmic pH and is eliminated at acidic pH [38]. As a result, NHE1 has been widely shown to play a role in cell cycle progression and cell proliferation. The role of NHE1 in cell proliferation was originally theorized based on its growth-promoting effect and was reckoned to be related to the mitogen-induced increase of pH_i_. It has been found that the role of NHE1 in ion translocation is crucial for conferring enhancement of proliferation [39]. The proliferation of NHE1-deficient cells is severely diminished, and the G2-M checkpoint is delayed [40]. However, it is not completely understood how NHE1 regulates cell proliferation. It has been demonstrated that an increase in pH_i_ may promote cell proliferation by facilitating protein synthesis. Concerning the role of cell volume, increases in cell volume and the uptake of inorganic ions are partially required for cell division, and this effect may be mediated by NHE1 [41]. NHE1 may impact cell proliferation by enhancing cell survival or suppressing apoptosis. Cell contraction and intracellular acidification are indications of apoptosis, whereas NHE1, increased regulatory volume, and intracellular alkalization may serve as an antiapoptotic signal [39]. The decrease in cytoplasmic volume results in NHE1-regulated Na^+^/H^+^ exchange, leading to phosphorylation and recruitment of ERM linkage to the cytosolic tail of NHE1. The interaction of ERM-NHE1 results in the development of a signaling complex, such as Akt and phosphatidylinositol 3-kinase (PI3K) which phosphorylates various substrates, contributing to the inhibition of apoptosis [42].

An intriguing area of research underway questions the processes of cell differentiation, some of which indicate the involvement of NHE1. Inhibition or deletion of NHE1 could impair the differentiation pathway [43]. It has been demonstrated that NHE1 activity promotes the differentiation of stem cells into the cardiomyocyte lineages. Elevated expression of NHE1 seems to have a role in promoting cardiomyocyte development [44]. NHE1 appears to either promote or prevent programmed cell death, depending on the cell type [45]. Furthermore, NHE1 has been assigned central roles in cytoskeletal tissue and cell motility. The cytoplasmic tail of NHE1 binds to ERM proteins and acts as an anchor for actin filaments. When these connections are disrupted or NHE1 activity is inhibited, external adhesion formation and cell migration are inhibited [40].

## 4. NHE1 and Myocardial Ischemia-Reperfusion Injury (MIRI)

### 4.1. Mechanisms of MIRI

The mechanisms associated with the pathogenesis of IRI are complicated, with the overall outcome of ischemia-induced perturbations and damage to all biomolecules in cells and tissues when the blood supply is re-established. When several pathological events occur concurrently, the injury is cumulative and the probability of irreversible myocardial injury increases significantly [46].

Several physiological mechanisms advance ischemia that results in hypoxia, including acute myocardial infarction and atherosclerosis. Hypoxia and malfunction of the electron transport chain in the mitochondria occur due to decreased arterial blood flow, which leads to ATP depletion and subsequent anaerobic glycolysis. Anaerobic metabolism, the only crucial source of new high-energy phosphate, produces less ATP and antioxidants in cells [47]. The deficiency of ATP during ischemia impairs the function of Na^+^-K^+^ ATPase, which causes Na^+^ retention inside the cells and K^+^ retention outside the cells. In cells with excessive Na^+^, NHE1 activity decreases. Also, lactic acid retention lowers pH_i_, contributing to the impairment of enzyme activity and the aggregation of nuclear chromatin. There is also a failure of Ca^2+^ pumps on the SR, which limits Ca^2+^ reuptake, thereby causing Ca^2+^ overload in cells [48]. In cells, the accumulation of H^+^, Na^+^, and Ca^2+^ leads to hypertonicity, resulting in the influx of water into the cytoplasm and cell swelling [49]. Furthermore, these alterations are concomitant with the opening of the mitochondrial permeability transition pore (PTP). Thus, the mitochondrial membrane potential is dissipated, further decreasing ATP synthesis [50]. If there is no rapid reperfusion of the coronary area and no collateral circulation, most myocardial hypoperfusion areas become necrotic. Prompt blood flow recovery can remove H^+^, accumulated in the extracellular space during ischemia, and provide oxygen and substrate for aerobic ATP production. On reperfusion, mitochondrial oxidative phosphorylase recovers to pre-ischemic levels within a few seconds, but contractility lags behind and only gradually reaches pre-ischemic levels [47]. Meanwhile, the production of reactive oxygen species (ROS) is increased because of the low concentration of antioxidants in ischemic cells. Mitochondrial damage and electrolyte imbalance during reperfusion enhance oxidative stress through the NADPH oxidase system, xanthine oxidase system, and uncoupled nitric oxide synthase system [51]. On the other hand, excessive ROS induces the release of pro-apoptotic factors through peroxidation of membrane lipids, which reduces membrane fluidity and increases Ca^2+^ permeability, exacerbating intracellular calcium overload and mitochondrial damage. Additionally, the inflammatory cascade and oxidative stress may trigger cytokine storms, leading to cell death resulting from structural impairment of cells [50].

### 4.2. Role of NHE1 in MIRI

During myocardial ischemia and reperfusion, the activity of NHE1 results in overall cell damage, which makes it an important target of drug intervention [45]. Inhibition of NHE1 is demonstrated as a potentially effective strategy to limit IRI. During ischemia, cardiomyocytes turn to anaerobic metabolism, producing protons that activate NHE1. When NHE1 is activated, it exchanges intracellular H^+^ for extracellular Na^+^, resulting in Na^+^ accumulation in cells. Na^+^ overload drives an increase in Ca^2+^ through the reversal of Na^+^/Ca^2+^ exchanger (NCX), thus leading to Ca^2+^ overload, ultimately triggering various pathways which cause cell death [52]. Evidence suggests that NHE1 inhibition during ischemia and reperfusion could protect the myocardium from Ca^2+^ overload [53]. Numerous investigations suggested protective effects on cardiac function of NHE inhibitors, particularly those selectively against NHE1 isoforms, such as amiloride, cariporide, and eniporide [54,55,56].

However, the mechanism for the elevation of intracellular Na^+^ concentration ([Na^+^]_i_) and when the exchanger functions during ischemia-reperfusion are somewhat controversial. During ischemia, elevated [Na^+^]_i_ was thought to be due to NHE1 activation, continuous Na^+^ channels, or the combination of both, while Na^+^ entry during reperfusion is principal via NHE1 [57]. In addition, metabolic status, which is affected by the degree of ischemia, also influences the increase of [Na^+^]_i_ during ischemia and reperfusion [14]. On the other hand, the question of whether the exchanger functions during ischemia-reperfusion is being debated. It has been reported that the accumulation of Na^+^ and Ca^2+^ in the ischemic non-reperfused myocardium is closely related to the ability of NHE1 inhibitors, which can attenuate these changes and reduce ischemia-induced injury and enhance functional recovery after reperfusion [58]. Using an animal model, the beneficial effects of the NHE1 inhibitor were more obvious when the drug was administered during the ischemic period than before reperfusion [59]. However, in a clinical trial conducted by Rupprecht et al. [60], cariporide provided protection even three weeks after percutaneous transluminal coronary angioplasty when administered directly before reperfusion. Nevertheless, the GUARDIAN [61] and EXPEDITION [62] trials support that the irreversible myocardial injury is relieved by NHE1 inhibitors administrated before ischemia. Moreover, several investigations have indicated that the myocardium is partially protected when NHE1 inhibitors are administered at the onset of reperfusion. Since Na^+^ appears to enter during both ischemia and reperfusion, it is assumed that the optimal therapeutic strategy includes continuous Na^+^ channel blockers, an NHE1 inhibitor during ischemia, and an NHE1 inhibitor during reperfusion [57].

## 5. NHE1 in Cardiac Hypertrophy and Heart Failure

### 5.1. Experimental Evidence for NHE1 Involvement in Cardiac Hypertrophy

Cardiac hypertrophy could be interpreted as a cardiac adaptation to the rise of hemodynamic loads caused by physiological stimulation (pregnancy, postpartum growth, and regular exercise) or pathological conditions (myocardial injury, hypertension, valvular heart disease, and neurohumoral hyperactivity) [63]. Therefore, cardiac hypertrophy could be categorized as physiological hypertrophy indicated by normal or strengthened contractility as well as normal cardiac structure [64], and pathological hypertrophy indicated by the decreased systolic and diastolic function of the heart. Pathological hypertrophy usually develops into heart failure after the discoordination between cardiomyocyte growth and cardiac angiogenesis [65]. Several studies have shown that pathological cardiac hypertrophy outweighs this adaptation as an independent risk contributor to heart failure and morbidity [66].

Cardiac hypertrophy is characterized by increased cell volume and the remodeling of the extracellular matrix (ECM) in cardiomyocytes. It has been suggested that the volume-regulated anion channel (VRAC), an essential factor in regulating cell volume, is associated with the progression of cardiac hypertrophy. Additionally, as a response to pathological stress, cardiomyocytes seem to undergo a transition from cell cycle-independent to cell cycle-dependent hypertrophic growth. NHE1 is also involved in the regulation of cell volume and cell cycle. Not surprisingly, much evidence suggests that NHE1 may represent a crucial downstream factor activated by various hypertrophic stimuli, especially after myocardial infarction. Cultured neonatal cardiomyocytes or isolated tissues were used to demonstrate the involvement of NHE1 in cardiac hypertrophy. Using vitro studies, the direct hypertrophic response to associated stimuli could be accurately studied, but the limitation of this approach is that the full picture of the sophisticated underlying mechanisms leading to the development of cardiac hypertrophy or heart failure couldn’t be resolved [67]. Nevertheless, the use of cultured neonatal cardiomyocytes provides important information for the understanding of the mechanisms of hypertrophic responses [68]. Studies using cardiomyocytes have consistently indicated that NHE1 inhibitors could prevent hypertrophic responses to a variety of stimuli, including angiotensin II (Ang II), endothelin 1(ET-1), phenylephrine, and aldosterone [69]. For example, elasticity-induced alkalinization of feline papillary muscles could be prevented by NHE1 inhibitors [70]. It has been shown that aldosterone stimulation induces hypertrophic responses in rat neonatal ventricular myocytes, accompanied by upregulation of NHE1 and elevation of [Na^+^]_i_, which could be prevented by NHE1-specific inhibitor EMD87580 [71]. NHE1 inhibitors might prevent hypertrophic responses to stimuli, indicating this antiporter is a downstream mediator. In vivo studies using related animal models have progressed the conception of NHE1-mediated cardiac hypertrophy and provided solid evidence for the involvement of this exchanger. Early studies have demonstrated that oral administration of NHE1 inhibitor amiloride decreases the fiber diameter in dilated cardiomyopathy mice and coronary artery ligation rats [72]. In N-line (wild-type NHE1) and K-line (active NHE1) transgenic models, activated NHE1 expression significantly increases interstitial fibrosis and cell apoptosis, and reduces cardiac performance, while expression of wild-type NHE1 results in more modest pathological changes. Mraiche et al. demonstrated that activated NHE1 is required to induce early myocardial hypertrophy in mice [73]. Additionally, it has been shown in vitro that the hyperactivity of NHE1 is adequate to generate Ca^2+^ signals required for cardiac hypertrophy occurrence [74]. Furthermore, silencing NHE1 specifically and locally in the left ventricle of spontaneous hypertensive rats (SHR) by L-shNHE1 (NHE1 with lentiviral delivery of small hairpin RNA) reduced the expression of NHE1 and the progression of left ventricular hypertrophy [66]. Therefore, NHE1 inhibition could be a novel approach to inducing regression of pathological cardiac hypertrophy.

### 5.2. Experimental Evidence for NHE1 Involvement in Heart Failure

Heart failure is a complex clinical syndrome induced by defective cardiac anatomy and/or function [3]. It is a common adverse effect of pathological cardiac hypertrophy. The hypertrophic response is matched with the increased workload during the compensatory phase of pathological cardiac hypertrophy, and therefore there is no negative effect on cardiac contractility. Conversely, the decompensated period of pathological cardiac hypertrophy follows this initial stage and results in contractile dysfunction and heart failure [75]. The activity of NHE1 has been proven to be remarkedly increased in animal models or patients with heart failure [76]. Therefore, NHE1 inhibition could serve as a therapeutic approach for treating heart failure.

The potential role of NHE1 in the development of heart failure was investigated using in vivo methods and heart failure models. The chronic rat model of coronary artery ligation is particularly useful because it’s characterized by a series of explicit post-infarction adaptive responses, which eventually contribute to heart failure, close to those in the clinical environment [68]. Utilizing this model, it has been demonstrated that oral administration of cariporide eliminates the increase in the overgrowth of surviving cardiomyocytes after one week of coronary artery occlusion and improves systolic dysfunction, which occurs without reducing afterload [77]. Kilić discovered that although the intervention of NHE1-specific inhibitors only occurs in the initial period after either administration of hypertrophic stimulation of cultured cardiomyocytes or coronary artery ligation, it has substantial benefits for developing myocardial hypertrophy and left ventricular dysfunction after coronary artery ligation [78]. Kusumoto et al. [79] have also demonstrated that NHE1 inhibition limits the early adaptive hypertrophic process to myocardial infarction and cardiac dysfunction in a rat model of myocardial infarction. In this model, the infarct size doesn’t change; however, the heart failure process is weakened. Moreover, these effects could be seen without any effect on blood pressure. Interestingly, it has been proposed that the activation of NHE1 in the heart and vascular system and of NHE3 in the kidney may be the common mechanism of diabetes mellitus and heart failure, and may also be the basis of their physiological overlap [80]. It was speculated that the mechanism of sodium-glucose co-transporter 2 (SGLT2) inhibitor-mediated cardioprotection is through an off-target effect on NHE1. It has been found that SGLT2 inhibitors (empagliflozin, dapagliflozin, and canagliflozin) could directly act on NHE1, possibly by binding to the Na^+^-binding site of NHE1, which lower [Na^+^]_i_ in the myocardium, inhibit the activity of NHE1, and thus produce physiological effects [81]. Studies suggest that reduced activity of NHE1 in the myocardium by SGLT2 inhibitors is related to the attenuation of oxidative stress, cardiac inflammation, fibrosis, autophagy, and ROS production [82,83,84]. As a result, inhibiting NHE1 may be a promising therapeutic approach for heart failure.

### 5.3. NHE1 Intracellular Signaling Regulates Cardiac Hypertrophy

Notably, G protein-coupled receptors, including the AngII, ET-1 and α_1_-adrenergic receptors, are considered deleterious in the progression of cardiac hypertrophy. The tandem arrangement of pathways is well understood: Gα_q_ activated NHE1 is mediated by a protein kinase C (PKC)-dependent mechanism, followed by Ca^2+^ mobilization and increased CaM [85]. Although there is emerging evidence for the involvement of NHE1 in the process of cardiac hypertrophy or heart failure and numerous possibilities have been put forward, the precise mechanism underlying the function of NHE1 remains to be determined. Despite NHE1-dependent pH_i_ changes that could be proposed considering the significance of pH in protein synthesis, the cytoplasmic alkalization induced by NHE1 could be negligible as other pH_i_-regulating membrane transporters would be recruited to maintain the physiological acid-base homeostasis [86].

The hyperactivity of NHE1 can lead to an increase in [Na^+^]_i_, which might be prevented by limiting NHE1 activity. The elevated [Na^+^]_i_ is considered to activate PKC isoenzymes, especially PKC_δ_ and PKC_ε_, and the reverse mode of NCX, thus increasing levels of intracellular Ca^2+^([Ca^2+^]_i_) [67]. Inhibitors of PKC could decrease the hypertrophic response, while cariporide reduced cardiac hypertrophy and PKC activation, thereby strengthening the link between NHE1 and PKC [68]. Increased [Ca^2+^]_i_, in turn, activates two significant Ca^2+^-dependent hypertrophic signaling pathways, Ca^2+^/CaM kinase II (CaMKII) and CaN [87,88].

CaMKII is a serine-threonine kinase that could phosphorylate many ion channels and Ca^2+^ transport proteins. CaMKII interacts with histone deacetylase (HDAC) when activated by Ca^2+^/CaM, which leads to the phosphorylation of HDAC and blockage of nuclear export or import; thus, HDAC-dependent target gene transcriptional suppression is blocked to promote cardiomyocyte hypertrophy [89,90]. It has been demonstrated that CaMKII could prevent cardiac hypertrophy through crosstalk with CaN [91]. Elevated [Ca^2+^]_i_ could be sensed by CaM, which activates CaN. CaN then dephosphorylates the nuclear factor of activated T cells (NFAT), thereby promoting the nuclear translocation of NFAT and activating hypertrophic gene transcription [92]. CaN also directly interacts with the specific sequence in the C-terminal domain of NHE1 to amplify the CaN/NFAT signaling pathway, enhancing the expression of hypertrophic genes [93]. These Ca^2+^-dependent hypertrophic molecules CaMKII and CaN are highly activated in NHE1 transgenic hearts [74]. In addition, increases in [Na^+^]_i_ have been assumed to activate salt-inducible kinase 1, which may directly affect the transcription of hypertrophic genes [94]. As such, NHE1-activated [Na^+^]_i_ and subsequent [Ca^2+^]_i_ increases appear to play a critical role in cardiac hypertrophy.

**Figure 2:**
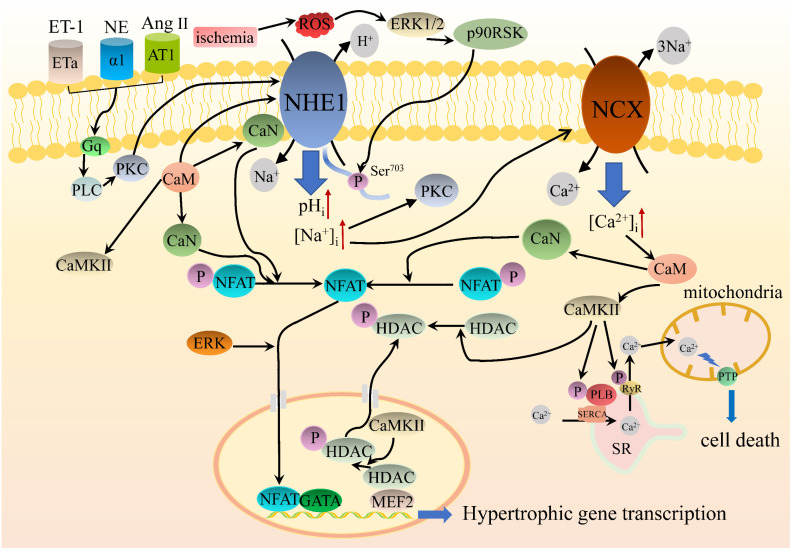
Diagrammatic representation of the main intracellular signaling pathways of NHE1 regulating cardiac hypertrophy. The activation of NHE1 by Gq protein-dependent phospholipase C (PLC) and subsequent Na^+^ overload is likely to be a crucial promotor of cardiac hypertrophy induced by various stimuli such as endothelin 1 (ET-1), norepinephrine (NE), and angiotensin II (Ang II) acting on their respective receptors. Increases in reactive oxygen species (ROS) production caused by cardiac ischemia may be involved in the activation of the ERK1/2-p90RSK pathway and therefore interact with the phosphorylation of serine 703. Ca^2+^/CaM activates NHE1 through direct interaction. NHE1 activation contributes to the increases of pH_i_ and [Na^+^]_i_. Increased [Na^+^]_i_ leads to sustained [Ca^2+^]_i_ elevation by acceleration of Ca^2+^ influx through the Na^+^/Ca^2+^ exchanger (NCX). Increased [Ca^2+^]_i_ activates two Ca^2+^-dependent hypertrophic signaling molecules, Ca^2+^/CaM-dependent kinase II (CaMKII) and calcineurin (CaN). Activated CaMKII mediates the phosphorylation of histone deacetylase (HDAC), relieving or derepressing myocyte enhancer factor 2 (MEF2), whereas CaN dephosphorylates the nuclear factor of activated T cells (NFAT) and interacts with the transcription factor GATA, promoting transcription of hypertrophic genes. On the other hand, activated CaMKII phosphorylates the phospholamban (PLB) to accelerate cytosolic Ca^2+^ uptake via the sarcoplasmic reticulum (SR) calcium transport ATPase (SERCA), and the ryanodine receptor (RyR) to increase SR Ca^2+^ release, thus augmenting the Ca^2+^ handling process. The increased SR Ca^2+^ leak could lead to mitochondrial Ca^2+^, which opens permeability transition pore (PTP) and ultimately results in cardiomyocyte apoptosis.

While it’s well established that elevated NHE1 activity plays a crucial role in heart failure, a potentially significant component of this indication is that NHE1 could activate pro-hypertrophic kinases, including Akt, extracellular signal-regulated kinase 1 and 2 (ERK1/2), and p90 ribosomal protein S6 kinase (p90RSK). Phenylephrine-induced activation of ERK1/2 and its downstream factor p90RSK have been demonstrated to induce the transcription of the hypertrophic gene in cardiomyocytes [95]. Furthermore, in guanyl cyclase-A deficient mice, a hypertrophic phenotype, elevated expression of NHE1 was related to increased cytosolic Ca^2+^ levels and concomitant activation of CaMKII and phosphorylation of p38, ERK1/2, and Akt [96]. NHE1 inhibition with cariporide remarkably ameliorated NHE1-induced heart failure, and only CaMKII and Akt activity were diminished, suggesting that CaMKII and Akt are critical mediators for NHE1-induced heart failure. In addition, it has been shown that some active molecules such as neurohormones, cytokines, and matricellular factors, are operative in heart failure. It has been reported that the increased expression of osteopontin (OPN) is found in conditions that promote myocardial hypertrophy and in an embryopathy model of type 2 diabetes mellitus, which induces cardiac fibrosis and hypertrophy. It has been demonstrated that overexpression of NHE1 contributes to upregulation of OPN, which induces cardiac hypertrophy via CD44 and p90RSK [87,97]. Recently, cathepsin B (Cat B) has been found to be involved in the anti-hypertrophic effect of NHE1. The release of Cat B into the ECM activates matrix metalloproteinase-9 (MMP-9). The active form of MMP-9 is expressed in the myocardium, and inhibition of which promotes cardiac hypertrophy through ERK and glycogen synthase 3β (GSK3β) signaling pathways [98]. NHE1 inhibition decreases the expression of Cat B and, in turn, prevents the activation of MMP-9, thereby protecting the myocardium from hypertrophy [99]. The potential intracellular signaling pathways of NHE1 regulating cardiac hypertrophy are shown in Figure 2.

## 6. Left Ventricular Hypertrophy (LVH) Leaves the Myocardium Susceptible to ISCHEMIA-Reperfusion Injury

LVH is causally associated with an increased risk of morbidity and mortality after acute myocardial infarction. Early studies have also suggested that LVH exacerbates myocardial necrosis and apoptosis during ischemia-reperfusion [100,101]. Nevertheless, little is known about the mechanism underlying the aggravated myocardial injury in hypertrophic hearts after myocardial infarction. Conditioning cardioprotective mechanisms seem to be intact or perhaps elevated in LVH before the onset of functional decompensation. Only if LVH progresses into a decompensated stage can the potential for protection be compromised [102]. Yano et al. [103] confirmed that hypertensive ventricular hypertrophy enhanced the susceptibility to necrosis and undermined cytoprotective signaling in spontaneously hypertensive stroke-prone rats (SHR-SPs). They demonstrated that increased production of ROS reduces the threshold for the opening of myocardial PTP, resulting in increased myocardial necrosis after ischemia-reperfusion. Mølgaard et al. [104] have verified that hearts from SHR-SPs with LVH are more vulnerable to ischemia-reperfusion, which seems to be reflected at the mitochondrial level by augmented damage to complexes III and IV in LVH hearts before the beginning of reperfusion. Furthermore, it has been demonstrated that LVH significantly exacerbates myocardial oxidative/nitrative stress in mice who succumbed to IRI. Scavenging superoxide or peroxynitrite could markedly ameliorate IRI in hypertrophied hearts, which is manifested by decreased myocardial oxidative/nitrative stress, attenuates myocardial infarction and impeded cardiomyocyte apoptosis. [101]. Since NHE1 inhibitors play a cardioprotective role in IRI, cardiac hypertrophy, heart failure, and ventricular hypertrophy enhance vulnerability to IRI, NHE1 inhibitors may serve as a potential drug target in cardiac hypertrophy and heart failure.

## 7. Clinical Perspectives

Multiple drugs such as amiloride, EIPA, zoniporide, and the more selective cariporide, eniporide, have been designed to inhibit NHE1 activity specifically [105]. These drugs exert their inhibitory effects by interacting with residues within TM4 and TM9 and reduce the activity of the exchanger by competing with Na^+^ [106]. Extensive data from preclinical studies suggest that pharmacological inhibition of NHE1 is cardioprotective, with the benefit being highest when administered before or shortly after the beginning of ischemia. Several animal models of heart failure, such as SHR, isolated cardiomyocytes, and transgenic models, have demonstrated that pharmacological inhibition of NHE1 can effectively prevent or induce the regression of cardiac remodeling. However, only a handful of studies examined the effects of NHE1 inhibitors in humans, and the translation of these positive preclinical data into the clinical arena has not been successful [107]. The ESCAMI (Evaluation of the Safety and Cardioprotective Effects of Eniporide in Acute Myocardial Infarction) [108] and GUARDIAN (GUARD During Ischemia Against Necrosis) studies [61] showed no clinical benefits. The EXPEDITION (Na^+^/H^+^ Exchange Inhibition to Prevent Coronary Events in Acute Cardiac Condition) study [62] paradoxically showed an increased incidence of cerebrovascular accidents and even mortality. In addition, there were also safety concerns, including the incidence of stroke and transient ischemic attacks with cariporide [62]. When administered for over two weeks, NHE1 inhibitors produced mild peripheral sensory axonal diseases [109]. Thus, despite favorable findings in animal models, large-scale clinical trials showed no benefits in patients with myocardial ischemia. Interestingly, a subset of patients with 150 mg eniporide and late reperfusion (>4 h) experienced a considerable reduction in the incidence of heart failure [108].

Rimeporide (initial code EMD87580) is a potent and selective inhibitor of NHE1. In hamsters with cardiomyopathy, it prevents cardiac hypertrophy, thrombosis, and necrosis and improves survival [110]. Recently, rimeporide was developed in clinical phase I for treating congestive heart failure and exhibited excellent safety and tolerability in adults [111]. The pathophysiological mechanisms of heart failure and Duchenne Muscular Dystrophy (DMD) overlap considerably. Therefore, NHE1 inhibition may be a promising therapeutical strategy for preventing the development of cardiomyopathy in DMD patients.

There are many reasons why clinical trials may fail, but one major reason could be that the animal models of the diseases on which the studies are based are excessively simplified. Most preclinical studies suggest that NHE1 inhibitors exhibit the greatest benefits when administered during ischemia and the initial phase of reperfusion. In animal studies, NHE1 inhibitors were given only at the onset of reperfusion, which allowed for better control than in clinical trials. Thus, it is perhaps not surprising that in a heterogeneous group of patients exhibiting varying degrees of coronary patency or possible myocardial necrosis. NHE-1 inhibitors given only at the reperfusion time have failed to show clinical efficacy in reducing infarct size. Allen concluded that large-scale and diversified trials might fail because few subjects have experienced ischemia; the post-infarction trial may fail because it failed to target the drug to crucial sites within the opportunity window, which seemed to begin instantly after reperfusion and lasted only 10–20 min [57]. Therefore, based on an in-depth analysis of the results obtained from these studies, it is rather simplistic and probably unreasonable to draw general statements about the failure of these clinical trials.

## 8. Conclusions

In this review, we described the mammalian NHE family and their physiological activities and highlighted the role of the widely studied protein subtype NHE1 in cardiac hypertrophy and heart failure. NHE1 is a ubiquitously expressed membrane transporter, known as a “housekeeping protein”, which regulates Na^+^-H^+^ transport, crucial for many cellular functions, such as maintaining pH_i_ and cell volume, regulating cell proliferation and apoptosis. In the myocardium, activated NHE1 leads to intracellular Na^+^ overload, subsequently increasing intracellular Ca^2+^ levels via NCX, ultimately resulting in Ca^2+^ overload and aggravation of cell injury. Substantial evidence suggests that NHE1 could serve as a downstream mediator to various hypertrophic stimuli, but the exact mechanisms for NHE1 involvement remain to be clarified. It has been demonstrated that activation of NHE1 can regulate the Ca^2+^-dependent prohypertrophic signaling pathways by altering [Na^+^]_i_ and pH_i_.

Furthermore, several NHE1 selective inhibitors have been developed, and the data from preclinical studies are quite inspiring. Unfortunately, the results obtained from clinical trials were rather complicated and generally unsatisfactory, so clinical trial data need to be extensively analyzed. Given the existing preclinical data demonstrating that pretreatment with NHE1 inhibitors or short-term use after hypotension can reduce cardiac dysfunction, clinical studies on patients with cardiac hypertrophy or heart failure need to confirm the optimal timing of drug administration. Besides, understanding the precise mechanism of NHE1 inhibition and the possible development of more specific and effective NHE1 inhibitors may be beneficial in this regard, and various other issues need to be addressed, such as the timing of administration of the inhibitors and the dosing levels of the inhibitors. Furthermore, it is necessary to monitor the complications encountered by patients in some clinical trials of myocardial infarction, mainly the slight increase in the prevalence of cerebrovascular diseases. Is NHE1 inhibition superior to other approaches, such as β receptor blockade or ACE inhibition? Do NHE1 inhibitors produce additive or synergistic effects in combination with other approaches to treat heart failure? Even though this novel therapeutic strategy seems difficult to practice at first glance, it is worth further investigation.

## Data Availability

Not applicable.

## References

[1] Tham Y.K., Bernardo B.C., Ooi J.Y., Weeks K.L., McMullen J.R. (2015). Pathophysiology of cardiac hypertrophy and heart failure: Signaling pathways and novel therapeutic targets. Arch. Toxicol..

[2] Yeves A.M., Ennis I.L. (2020). Na^+^/H^+^ exchanger and cardiac hypertrophy. Hipertens. Riesgo. Vasc..

[3] Choi H.M., Park M.S., Youn J.C. (2019). Update on heart failure management and future directions. Korean J. Intern. Med..

[4] Di Palo K.E., Barone N.J. (2020). Hypertension and Heart Failure. Heart Fail. Clin..

[5] Hammond G., Rich M.W. (2019). Hypertensive Heart Failure in the Very Old. Heart. Fail. Clin..

[6] Agra Bermejo R.M., Gonzalez Ferreiro R., Varela Roman A., Gomez Otero I., Kreidieh O., Conde Sabaris P., Rodriguez-Manero M., Moure Gonzalez M., Seoane Blanco A., Virgos Lamela A. (2017). Nutritional status is related to heart failure severity and hospital readmissions in acute heart failure. Int. J. Cardiol..

[7] Yasuhara S., Maekawa M., Bamba S., Kurihara M., Nakanishi N., Yamamoto T., Sakai H., Yagi N., Nakagawa Y., Sasaki M. (2020). Energy Metabolism and Nutritional Status in Hospitalized Patients with Chronic Heart Failure. Ann. Nutr. Metab..

[8] von Haehling S., Doehner W., Anker S.D. (2007). Nutrition, metabolism, and the complex pathophysiology of cachexia in chronic heart failure. Cardiovasc. Res..

[9] Giovannini S., Onder G., Lattanzio F., Bustacchini S., Di Stefano G., Moresi R., Russo A., Bernabei R., Landi F. (2018). Selenium concentrations and mortality among community-dwelling older adults: Results from ilSIRENTE. J. Nutr. Health Aging.

[10] Krishnaswami A., Steinman M.A., Goyal P., Zullo A.R., Anderson T.S., Birtcher K.K., Goodlin S.J., Maurer M.S., Alexander K.P., Rich M.W. (2019). Deprescribing in Older Adults With Cardiovascular Disease. J. Am. Coll. Cardiol..

[11] Georgiev K.D., Hvarchanova N., Georgieva M., Kanazirev B. (2019). The role of the clinical pharmacist in the prevention of potential drug interactions in geriatric heart failure patients. Int. J. Clin. Pharm..

[12] Tanai E., Frantz S. (2015). Pathophysiology of Heart Failure. Compr. Physiol..

[13] Basso C., Thiene G. (2006). The pathophysiology of myocardial reperfusion: A pathologist’s perspective. Heart.

[14] Kalogeris T., Baines C.P., Krenz M., Korthuis R.J. (2016). Ischemia/Reperfusion. Compr. Physiol..

[15] Wan Ab Naim W.N., Mohamed Mokhtarudin M.J., Chan B.T., Lim E., Ahmad Bakir A., Nik Mohamed N.A. (2021). The study of myocardial ischemia-reperfusion treatment through computational modelling. J. Theor. Biol..

[16] Avkiran M., Marber M.S. (2002). Na^+^/H^+^ exchange inhibitors for cardioprotective therapy: Progress, problems and prospects. J. Am. Coll. Cardiol..

[17] Gurney M.A., Laubitz D., Ghishan F.K., Kiela P.R. (2017). Pathophysiology of intestinal Na^+^/H^+^ exchange. Cell Mol. Gastroenterol. Hepatol..

[18] Escudero D.S., Perez N.G., Diaz R.G. (2021). Myocardial Impact of NHE1 Regulation by Sildenafil. Front. Cardiovasc. Med..

[19] Mithell P., Moyle J. (1967). Acid-Base titration across the membrane system of rat-liver mitochondria. Biochem. J..

[20] Parker M.D., Myers E.J., Schelling J.R. (2015). Na^+^-H^+^ exchanger-1 (NHE1) regulation in kidney proximal tubule. Cell Mol. Life Sci..

[21] Donowitz M., Ming Tse C., Fuster D. (2013). SLC9/NHE gene family, a plasma membrane and organellar family of Na^+^/H^+^ exchangers. Mol. Asp. Med..

[22] Hendus-Altenburger R., Kragelund B.B., Pedersen S.F. (2014). Structural dynamics and regulation of the mammalian SLC9A family of Na^+^/H^+^ exchangers. Curr. Top. Membr..

[23] Lee S.H., Kim T., Park E.S., Yang S., Jeong D., Choi Y., Rho J. (2008). NHE10, an osteoclast-specific member of the Na^+^/H^+^ exchanger family, regulates osteoclast differentiation and survival. Biochem. Biophys. Res. Commun..

[24] Fliegel L. (2019). Structural and functional changes in the Na^+^/H^+^ exchanger isoform 1, induced by Erk1/2 phosphorylation. Int. J. Mol. Sci..

[25] Sardet C., Franchi A., Pouysségur J. (1989). Molecular cloning, primary structure, and expression of the human growth factor-activatable Na^+^/H^+^ antiporter. Cell.

[26] Shrode L.D., Gan B.S., D’Souza S.J., Orlowski J., Grinstein S. (1998). Topological analysis of NHE1, the ubiquitous Na^+^/H^+^ exchanger using chymotryptic cleavage. Am. J. Physiol. Cell Physiol..

[27] Wakabayashi S., Pang T., Su X., Shigekawa M. (2000). A novel topology model of the human Na^+^/H^+^ exchanger isoform 1. J. Biol. Chem..

[28] Li T., Tuo B. (2020). Pathophysiology of hepatic Na+/H+ exchange (Review). Exp. Ther. Med..

[29] Dutta D., Fliegel L. (2019). Molecular modeling and inhibitor docking analysis of the Na^+^/H^+^ exchanger isoform one. Biochem. Cell Biol..

[30] Landau M., Herz K., Padan E., Ben-Tal N. (2007). Model structure of the Na^+^/H^+^ exchanger 1 (NHE1): Functional and clinical implications. J. Biol. Chem..

[31] Lee B.L., Sykes B.D., Fliegel L. (2011). Structural analysis of the Na^+^/H^+^ exchanger isoform 1 (NHE1) using the divide and conquer approach. Biochem. Cell Biol..

[32] Liu Y., Basu A., Li X., Fliegel L. (2015). Topological analysis of the Na^+^/H^+^ exchanger. Biochim. Biophys. Acta.

[33] Fliegel L. (2021). Role of genetic mutations of the Na^+^/H^+^ exchanger isoform 1, in human isease and protein targeting and activity. Mol. Cell Biochem..

[34] Orlowski J., Grinstein S. (2004). Diversity of the mammalian sodium/proton exchanger SLC9 gene family. Pflug. Arch..

[35] Orlowski J., Grinstein S. (2011). Na^+^/H^+^ exchangers. Compr. Physiol..

[36] Alexander R.T., Grinstein S. (2006). Na^+^/H^+^ exchangers and the regulation of volume. Acta Physiol..

[37] Hoffmann E.K., Pedersen S.F. (2011). Cell volume homeostatic mechanisms: Effectors and signalling pathways. Acta Physiol..

[38] Putney L.K., Barber D.L. (2004). Expression profile of genes regulated by activity of the Na-H exchanger NHE1. BMC Genom..

[39] Putney L.K., Denker S.P., Barber D.L. (1999). The changing face of the Na^+^/H^+^ exchanger, NHE1: Structure, regulation, and cell actions. Annu. Rev. Pharmacol. Toxicol..

[40] Fliegel L. (2005). The Na^+^/H^+^ exchanger isoform 1. Int. J. Biochem. Cell Biol..

[41] Pedersen S.F. (2006). The Na^+^/H^+^ exchanger NHE1 in stress-induced signal transduction: Implications for cell proliferation and cell death. Pflug. Arch..

[42] Schelling J.R., Abu Jawdeh B.G. (2008). Regulation of cell survival by Na^+^/H^+^ exchanger-1. Am. J. Physiol. Ren. Physiol..

[43] Pedersen S.F., Counillon L. (2019). The SLC9A-C mammalian Na^+^/H^+^ exchanger family: Molecules, mechanisms, and physiology. Physiol. Rev..

[44] Li X., Karki P., Lei L., Wang H., Fliegel L. (2009). Na^+^/H^+^ exchanger isoform 1 facilitates cardiomyocyte embryonic stem cell differentiation. Am. J. Physiol. Heart Circ. Physiol..

[45] Malo M.E., Fliegel L. (2006). Physiological role and regulation of the Na^+^/H^+^ exchanger. Can. J. Physiol. Pharmacol..

[46] Martins G.F., Martins G. (2017). Role of trimetazidine in coronary artery bypass graft surgery. World J. Cardiovasc. Surg..

[47] Frank A., Bonney M., Bonney S., Weitzel L., Koeppen M., Eckle T. (2012). Myocardial ischemia reperfusion injury: From basic science to clinical bedside. Semin. Cardiothorac. Vasc. Anesth..

[48] Kalogeris T., Baines C.P., Krenz M., Korthuis R.J. (2012). Cell biology of ischemia/reperfusion injury. Int. Rev. Cell Mol. Biol..

[49] Rout A., Tantry U.S., Novakovic M., Sukhi A., Gurbel P.A. (2020). Targeted pharmacotherapy for ischemia reperfusion injury in acute myocardial infarction. Expert Opin. Pharm..

[50] Wu M.Y., Yiang G.T., Liao W.T., Tsai A.P., Cheng Y.L., Cheng P.W., Li C.Y., Li C.J. (2018). Current mechanistic concepts in ischemia and reperfusion injury. Cell Physiol. Biochem..

[51] Fischesser D.M., Bo B., Benton R.P., Su H., Jahanpanah N., Haworth K.J. (2021). Controlling reperfusion injury with controlled reperfusion: Historical perspectives and new paradigms. J. Cardiovasc. Pharmacol. Ther..

[52] Lu M., Jia M., Wang Q., Guo Y., Li C., Ren B., Qian F., Wu J. (2021). The electrogenic sodium bicarbonate cotransporter and its roles in the myocardial ischemia-reperfusion induced cardiac diseases. Life Sci..

[53] Fliegel L. (2009). Regulation of the Na^+^/H^+^ exchanger in the healthy and diseased myocardium. Expert Opin. Ther. Targets.

[54] Karmazyn M. (2000). Pharmacology and clinical assessment of cariporide for the treatment coronary artery diseases. Exp. Opin. Investig. Drugs.

[55] Rabkin D.G., Cabreriza S.E., Cheema F.H., Hill A.A., Curtis L.J., Sciacca R.R., Mosca R.S., Spotnitz H.M. (2003). Cariporide is cardioprotective after iatrogenic ventricular fibrillation in the intact swine heart. Ann. Thorac. Surg..

[56] Wang Y., Meyer J.W., Ashraf M., Shull G.E. (2003). Mice with a null mutation in the NHE1 Na^+^-H^+^ exchanger are resistant to cardiac ischemia-reperfusion injury. Circ. Res..

[57] Murphy E., Allen D.G. (2009). Why did the NHE inhibitor clinical trials fail?. J. Mol. Cell. Cardiol..

[58] Gazmuri R.J., Radhakrishnan J., Ayoub I.M. (2019). Sodium-hydrogen exchanger isoform-1 inhibition: A promising pharmacological intervention for resuscitation from cardiac arrest. Molecules.

[59] Klein H.H., Pich S., Bohle R.M., Lindert-Heimberg S., Nebendahl K. (2000). Na^+^/H^+^ exchange inhibitor cariporide attenuates cell injury predominantly during ischemia and not at onset of reperfusion in porcine hearts with low residual blood flow. Circulation.

[60] Rupprecht H.J., Dahl J.V., Terres W., Seyfarth K.M., Richardt G., Schultheiβ H.P., Sheehan F.H., Drexler H. (2000). Cardioprotective effects of the Na^+^/H^+^ exchange inhibitor cariporide in patients with acute anterior myocardial infarction undergoing direct PTCA. Circulation.

[61] Boyce S.W., Bartels C., Bolli R., Chaitman B., Chen J.C., Chi E., Jessel A., Kereiakes D., Knight J., Thulin L. (2003). Impact of sodium-hydrogen exchange inhibition by cariporide on death or myocardial infarction in high-risk CABG surgery patients: Results of the CABG surgery cohort of the GUARDIAN study. J. Thorac. Cardiov. Surg..

[62] Mentzer R.M., Bartels C., Bolli R., Boyce S., Buckberg G.D., Chaitman B., Haverich A., Knight J., Menasche P., Myers M.L. (2008). Sodium-hydrogen exchange inhibition by cariporide to reduce the risk of ischemic cardiac events in patients undergoing coronary artery bypass grafting: Results of the EXPEDITION study. Ann. Thorac. Surg..

[63] Catalucci D., Latronico M.V.G., Ellingsen O., Condorelli G. (2008). Physiological myocardial hypertrophy: How and why?. Front. Biosci..

[64] Weeks K.L., McMullen J.R. (2011). The athlete’s heart vs. the failing heart: Can signaling explain the two distinct outcomes?. Physiology.

[65] Shimizu I., Minamino T. (2016). Physiological and pathological cardiac hypertrophy. J. Mol. Cell. Cardiol..

[66] Nolly M.B., Pinilla A.O., Ennis I.L., Cingolani H.E., Morgan P.E. (2015). Cardiac hypertrophy reduction in SHR by specific silencing of myocardial Na^+^/H^+^ exchanger. J. Appl. Physiol..

[67] Fliegel L., Karmazyn M. (2004). The cardiac Na-H exchanger: A key downstream mediator for the cellular hypertrophic effects of paracrine, autocrine and hormonal factors. Biochem. Cell Biol..

[68] Karmazyn M. (2001). Therapeutic potential of Na-H exchange inhibitors for the treatment of heart failure. Expert Opin. Investig. Drugs.

[69] Suleiman M., Abdulrahman N., Yalcin H., Mraiche F. (2018). The role of CD44, hyaluronan and NHE1 in cardiac remodeling. Life Sci..

[70] Cingolani H.E., Alvarez B.V., Ennis I.L., Camilión de Hurtado M.C. (1998). Stretch-induced alkalinization of feline papillary muscle an autocrine-paracrine system. Circ. Res..

[71] Karmazyn M., Liu Q., Gan X.T., Brix B.J., Fliegel L. (2003). Aldosterone increases NHE-1 expression and induces NHE-1-dependent hypertrophy in neonatal rat ventricular myocytes. Hypertension.

[72] Hasegawa S., Nakano M., Taniguchi Y., Imai S., Murata K., Suzuki T. (1995). Effects of Na^+^-H^+^ exchange blocker amiloride on left ventricular remodeling after anterior myocardial infarction in rats. Cardiovasc. Drug Ther..

[73] Mraiche F., Oka T., Gan X.T., Karmazyn M., Fliegel L. (2011). Activated NHE1 is required to induce early cardiac hypertrophy in mice. Basic Res. Cardiol..

[74] Nakamura T.Y., Iwata Y., Arai Y., Komamura K., Wakabayashi S. (2008). Activation of Na^+^/H^+^ exchanger 1 is sufficient to generate Ca^2+^ signals that induce cardiac hypertrophy and heart failure. Circ. Res..

[75] Rosca M.G., Tandler B., Hoppel C.L. (2013). Mitochondria in cardiac hypertrophy and heart failure. J. Mol. Cell. Cardiol..

[76] Baartscheer A., Hardziyenka M., Schumacher C.A., Belterman C.N., van Borren M.M., Verkerk A.O., Coronel R., Fiolet J.W. (2008). Chronic inhibition of the Na^+^/H^+^ exchanger causes regression of hypertrophy, heart failure, and ionic and electrophysiological remodelling. Br. J. Pharmacol..

[77] Chen L., Chen X.C., Gan X.T., Beier N., Scholz W., Karmazyn M. (2004). Inhibition and reversal of myocardial infarctioninduced hypertrophy and heart failure by NHE-1 inhibition. Am. J. Physiol. Heart Circ. Physiol..

[78] Kilić A., Huang C.X., Rajapurohitam V., Madwed J.B., Karmazyn M. (2014). Early and transient sodium-hydrogen exchanger isoform 1 inhibition attenuates subsequent cardiac hypertrophy and heart failure following coronary artery ligation. J. Pharmacol. Exp. Ther..

[79] Kusumoto K., Haist J.V., Karmazyn K. (2001). Na^+^/H^+^ exchange inhibition reduces hypertrophy and heart failure after myocardial infarction in rats. Am. J. Physiol. Heart Circ. Physiol..

[80] Packer M. (2017). Activation and inhibition of sodium-hydrogen exchanger is a mechanism that links the pathophysiology and treatment of diabetes Mmellitus with that of heart failure. Circulation.

[81] Despa S. (2018). Myocyte [Na^+^]_i_ dysregulation in heart failure and diabetic cardiomyopathy. Front. Physiol..

[82] Filippatos T.D., Liontos A., Papakitsou I., Elisaf M.S. (2019). SGLT2 inhibitors and cardioprotection: A matter of debate and multiple hypotheses. Postgrad. Med..

[83] Yu Y.W., Que J.Q., Liu S., Huang K.Y., Qian L., Weng Y.B., Rong F.N., Wang L., Zhou Y.Y., Xue Y.J. (2021). Sodium-Glucose Co-transporter-2 Inhibitor of Dapagliflozin Attenuates Myocardial Ischemia/Reperfusion Injury by Limiting NLRP3 Inflammasome Activation and Modulating Autophagy. Front. Cardiovasc. Med..

[84] Li X., Romer G., Kerindongo R.P., Hermanides J., Albrecht M., Hollmann M.W., Zuurbier C.J., Preckel B., Weber N.C. (2021). Sodium Glucose Co-Transporter 2 Inhibitors Ameliorate Endothelium Barrier Dysfunction Induced by Cyclic Stretch through Inhibition of Reactive Oxygen Species. Int. J. Mol. Sci..

[85] Linz W.J., Busch A.E. (2003). NHE-1 inhibition: From protection during acute ischaemia/reperfusion to prevention/reversal of myocardial remodelling. Naunyn-Schmiedeberg’s Arch. Pharmacol..

[86] Karmazyn M., Sawyer M., Fliegel L. (2005). The Na^+^/H^+^ exchanger: A target for cardiac therapeutic intervention. Curr. Drug Targets-Cardiovasc. Hematol. Disord..

[87] Mohamed I.A., Mraiche F. (2015). Targeting osteopontin, the silent partner of Na^+^/H^+^ exchanger isoform 1 in cardiac remodeling. J. Cell Physiol..

[88] Wakabayashi S., Hisamitsu T., Nakamura T.Y. (2013). Regulation of the cardiac Na^+^/H^+^ exchanger in health and disease. J. Mol. Cell. Cardiol..

[89] Backs J., Song K., Bezprozvannaya S., Chang S., Olson E.N. (2006). CaM kinase II selectively signals to histone deacetylase 4 during cardiomyocyte hypertrophy. J. Clin. Investig..

[90] Anderson M.E., Brown J.H., Bers D.M. (2011). CaMKII in myocardial hypertrophy and heart failure. J. Mol. Cell. Cardiol..

[91] Backs J., Backs T., Neef S., Kreusser M., Lehmann L.H., Patrick D.M., Grueter C.E., Qi X., Richardson J.A., Hill J.A. (2009). The δ isoform of CaM kinase II is required for pathological cardiac hypertrophy and remodeling after pressure overload. Proc. Natl. Acad. Sci. USA.

[92] Samak M., Fatullayev J., Sabashnikov A., Zeriouh M., Schmack B., Farag M., Popov A.F., Dohmen P.M., Choi Y.H., Wahlers T. (2016). Cardiac hypertrophy: An introduction to molecular and cellular basis. Med. Sci. Monit. Basic Res..

[93] Hisamitsu T., Nakamura T.Y., Wakabayashi S. (2012). Na^+^/H^+^ exchanger 1 directly binds to calcineurin A and activates downstream NFAT signaling, leading to cardiomyocyte hypertrophy. Mol. Cell Biol..

[94] Popov S., Venetsanou K., Chedrese P.J., Pinto V., Takemori H., Franco-Cereceda A., Eriksson P., Mochizuki N., Soares-da-Silva P., Bertorello A.M. (2012). Increases in intracellular sodium activate transcription and gene expression via the salt-inducible kinase 1 network in an atrial myocyte cell line. Am. J. Physiol. Heart Circ. Physiol..

[95] Amirak E., Fuller S.J., Sugden P.H., Clerk A. (2013). p90 ribosomal S6 kinases play a significant role in early gene regulation in the cardiomyocyte response to G_q_-protein-coupled receptor stimuli, endothelin-1 and α_1_-adrenergic receptor agonists. Biochem. J..

[96] Kilić A., Velic A., De Windt L.J., Fabritz L., Voss M., Mitko D., Zwiener M., Baba H.A., van Eickels M., Schlatter E. (2005). Enhanced activity of the myocardial Na^+^/H^+^ exchanger NHE-1 contributes to cardiac remodeling in atrial natriuretic peptide receptor-deficient mice. Circulation.

[97] Abdulrahman N., Jaspard-Vinassa B., Fliegel L., Jabeen A., Riaz S., Gadeau A.P., Mraiche F. (2018). Na^+^/H^+^ exchanger isoform 1-induced osteopontin expression facilitates cardiac hypertrophy through p90 ribosomal S6 kinase. Physiol. Genom..

[98] Euler G., Locquet F., Kociszewska J., Osygus Y., Heger J., Schreckenberg R., Schlüter K.D., Kenyeres É., Szabados T., Bencsik P. (2021). Matrix metalloproteinases repress hypertrophic growth in cardiac myocytes. Cardiovasc. Drug Ther..

[99] Riaz S., Abdulrahman N., Uddin S., Jabeen A., Gadeau A.P., Fliegel L., Mraiche F. (2020). Anti-hypertrophic effect of Na^+^/H^+^ exchanger-1 inhibition is mediated by reduced cathepsin B. Eur. J. Pharmacol..

[100] Dellsperger K.C., Clothier J.L., Hartnett J.A., Haun L.M., Marcus M.L. (1988). Acceleration of the wavefront of myocardial necrosis by chronic hypertension and left ventricular hypertrophy in dogs. Circ. Res..

[101] Ma L.L., Li Y., Yin P.P., Kong F.J., Guo J.J., Shi H.T., Zhu J.B., Zou Y.Z., Ge J.B. (2018). Hypertrophied myocardium is vulnerable to ischemia/reperfusion injury and refractory to rapamycin-induced protection due to increased oxidative/nitrative stress. Clin. Sci..

[102] Pagliaro P., Penna C. (2017). Hypertension, hypertrophy, and reperfusion injury. J. Cardiovasc. Med..

[103] Yano T., Miki T., Tanno M., Kuno A., Itoh T., Takada A., Sato T., Kouzu H., Shimamoto K., Miura T. (2011). Hypertensive hypertrophied myocardium is vulnerable to infarction and refractory to erythropoietin-induced protection. Hypertension.

[104] Molgaard S., Faricelli B., Salomonsson M., Engstrom T., Treiman M. (2016). Increased myocardial vulnerability to ischemia-reperfusion injury in the presence of left ventricular hypertrophy. J. Hypertens..

[105] Madonna R., De Caterina R. (2013). Sodium-hydrogen exchangers (NHE) in human cardiovascular diseases: Interfering strategies and their therapeutic applications. Vasc. Pharmacol..

[106] Wu D., Kraut J.A. (2011). Potential role of NHE1 (sodium-hydrogen exchanger 1) in the cellular dysfunction of lactic acidosis: Implications for treatment. Am. J. Kidney Dis..

[107] Karmazyn M. (2013). NHE-1: Still a viable therapeutic target. J. Mol. Cell. Cardiol..

[108] Zeymer U., Suryapranata H., Monassier J.P., Opolski G., Davies J., Rasmanis G., Linssen G., Tebble U., Schröder R., Tiemann R. (2001). The Na^+^/H^+^ exchange inhibitor eniporide as an adjunct to early reperfusion therapy for acute myocardial infarction. J. Am. Coll. Cardiol..

[109] Pettersen J.C., Chouinard L., Kerlin R.L., Groom S.N., Botts S., Arezzo J.C., Boucher M.A., Frazier D.E., Buchholz A.R. (2008). Neurotoxic effects of zoniporide: A selective inhibitor of the Na^+^/H^+^ exchanger isoform 1. Toxicol. Pathol..

[110] Thomé F.P., Su J., Barthelemy I., Blanchard-Gutton N., Bkaily G., Yu Q., Nagaraju K., Galeh B., Blot S. (2016). Translational development of rimeporide, a sodium-hydrogen exchanger (NHE-1) inhibitor, for patients with Duchenne muscular dystrophy. Neuromuscul. Disord..

[111] Previtali S.C., Gidaro T., Diaz-Manera J., Zambon A., Carnesecchi S., Roux-Lombard P., Spitali P., Signorelli M., Szigyarto C.A., Johansson C. (2020). Rimeporide as a first-in-class NHE-1 inhibitor: Results of a phase Ib trial in young patients with Duchenne Muscular Dystrophy. Pharmacol. Res..

